# Sale of Horse-Flesh as Food

**Published:** 1857-10

**Authors:** 


### SALE OF HORSE-FLESH AS FOOD.

Our respected contemporary, the *Veterinarian*, has an account in the September number, of a visit to the shop of a horse-butcher at Altona (Holstein). The writer, who was making a continental tour, saw exposed for sale the hind quarters and sundry pieces of the flesh of a horse. There were four other establishments of the same kind in the town. The meat was said to have a ready sale at from 2*d.* to 3*d.*, English money, per pound. The butcheries are licensed by the Government, and are under the supervision of the police. Notice has to be given before a horse can be killed, when the department veterinary surgeon attends and examines the animal; and, if it is found free from constitutional disease, notwithstanding it may be incapacitated for work from lameness or other defects, he certifies to that effect, and for the sake of identity brands the animal on its hoof. Within a given time the animal must be killed, and its leg and foot produced for the inspection and satisfaction of the police. It is said that the meat is often bought by persons who cannot properly be said to belong to the lower classes.

